# Extensive sensorimotor training enhances nociceptive cortical responses in healthy individuals

**DOI:** 10.1002/ejp.2057

**Published:** 2022-12-01

**Authors:** Anna M. Zamorano, Boris Kleber, Federico Arguissain, Peter Vuust, Herta Flor, Thomas Graven‐Nielsen

**Affiliations:** ^1^ Center for Neuroplasticity and Pain (CNAP), Department of Health Science and Technology Aalborg University Aalborg Denmark; ^2^ Center for Music in the Brain, Dept. of Clinical Medicine Aarhus University & The Royal Academy of Music Aarhus/Aalborg Aarhus and Aalborg Denmark; ^3^ Department of Cognitive and Clinical Neuroscience, Central Institute of Mental Health, Medical Faculty Mannheim University of Heidelberg Mannheim Germany

## Abstract

**Background:**

Prolonged and repeated sensorimotor training is a crucial driver for promoting use‐dependent plasticity, but also a main risk factor for developing musculoskeletal pain syndromes, yet the neural underpinnings that link repetitive movements to abnormal pain processing are unknown.

**Methods:**

Twenty healthy musicians, one of the best in vivo models to study use‐dependent plasticity, and 20 healthy non‐musicians were recruited. Perceptual thresholds, reaction times (RTs) and event‐related potentials (ERPs) were recorded using nociceptive intra‐epidermal and non‐nociceptive transcutaneous electrical stimulation.

**Results:**

In response to comparable stimulus intensities, musicians compared to non‐musicians showed larger non‐nociceptive N140 (associated with higher activation of regions within the salience network), higher nociceptive N200 ERPs (associated with higher activation of regions within the sensorimotor network) and faster RTs to both stimuli. Non‐musicians showed larger non‐nociceptive P200 ERP. Notably, a similar P200 component prominently emerged during nociceptive stimulation in non‐musicians. Across participants, larger N140 and N200 ERPs were associated with RTs, whereas the amount of daily practice in musicians explained non‐nociceptive P200 and nociceptive P300 ERPs.

**Conclusions:**

These novel findings indicate that the mechanisms by which extensive sensorimotor training promotes use‐dependent plasticity in multisensory neural structures may also shape the neural signatures of nociceptive processing in healthy individuals.

**Significance:**

Repetitive sensorimotor training may increase the responsiveness of nociceptive evoked potentials. These novel data highlight the importance of repetitive sensorimotor practice as a contributing factor to the interindividual variability of nociceptive‐related potentials.

## 1 INTRODUCTION

Repetitive movements and their associated multisensory integration (i.e. sensorimotor training) play a major role in the structural and functional reorganization of sensory and motor neural connections (Bütefisch et al., 2000; Classen et al., 1998; Recanzone et al., 1993; Zatorre et al., 2012). This phenomenon, known as use‐dependent plasticity, has been widely investigated in experienced musicians, as musical training is a popular in vivo model for evaluating the effects of extensive sensorimotor training (Herholz & Zatorre, 2012; Jäncke, 2009) and has been associated with functional and structural adaptive changes in brain regions involved in sensory perception (Kraus & Chandrasekaran, 2010; Pantev et al., 1998), sensorimotor control (Kleber et al., 2013; Rosenkranz et al., 2007) and cognitive functions (Brown et al., 2015). Despite such adaptive effects, animal models have shown that extensive sensorimotor training can also contribute to the genesis of maladaptive neural plasticity assumed to be involved in the development of pain syndromes and focal dystonia (Byl et al., 1996). In humans, the prolonged and repeated execution of motor patterns is also considered one of the main risk factors for developing pain musculoskeletal syndromes (Herin et al., 2014), yet the neural underpinnings that link repetitive movements to abnormal pain processing are still unknown.

In order to determine the neural foundation by which extensive sensorimotor training may alter nociceptive processing in humans, the current study directly explored the nociceptive and non‐nociceptive somatosensory pathways in healthy individuals who perform extensive repetitive movements (i.e., trained musicians). The underlying hypothesis is that the same processes by which extensive sensorimotor training and multisensory integration can modify task‐specific topographic and functional neural representations in the brain (Byl et al., 1996; Elbert et al., 1995), as well as facilitate the priming of neural responses in brain areas where the processing of multimodal stimuli converges (Paraskevopoulos et al., 2012), may also shape nociceptive neural and behavioural responses, as previously indicated by invertebrate models (Hu et al., 2017; Ohyama et al., 2015). Nociceptive inputs are conveyed via the spinothalamic pathways to the brain, engaging multiple regions such as the primary and secondary somatosensory cortices, the prefrontal cortex, the insula, and the anterior cingulate cortex, as well as subcortical areas like the thalamus (Apkarian et al., 2005). Likewise, non‐nociceptive inputs, which commonly convey via the dorsal column–lemniscal pathways, can also converge in the nociceptive pathways by gaining access to wide dynamic range (WDR) neurons in the spinal cord (D'Mello & Dickenson, 2008). Based on this notion and our observations of increased pain sensitivity and insula connectivity in healthy individuals performing extensive motor training (Zamorano et al., 2015, 2017, 2019), we propose that repetitive sensorimotor training and multimodal integration can also prime the insular and cingulate responses to other sensory processes, such as nociception.

To test this assumption, we used nociceptive and non‐nociceptive electrical stimulation paradigms to assess the spinothalamic and the dorsal column–lemniscal pathways with the aim of understanding whether extensive sensorimotor training in healthy individuals can facilitate the transient brain responses as well as higher brain activation. By analysing the neural and behavioural response components as a function of accumulated sensorimotor training (i.e. musical practice), we also aimed to characterize how extensive multisensory training may influence the variability of cortical responses to noxious and non‐noxious stimuli across individuals. Following our hypotheses, we expected enhanced nociceptive and non‐nociceptive evoked responses in healthy musicians relative to non‐musicians. Moreover, we expected that the amount of sensorimotor musical training would be associated with the variation of the evoked brain responses and reaction times.

## 2 MATERIALS AND METHODS

### 2.1 Participants

Twenty trained musicians (nine female, 18 right‐handed, mean age 26.5 ± 3.8 years), consisting of 6 strings, 3 keyboards, 8 woodwinds and 3 brass players, participated in this study. In addition to their main instrument, 13 out of 20 musicians occasionally played a second instrument (6 keyboards, 2 strings, 2 woodwinds, 2 voices and 1 brass instrument). All musicians were conservatory‐trained instrumentalists. Their average age of onset with musical training of 8.3 years (±2.6), leading up to extensive professional experience: a total average of 18,102 h (±8322 h) of musical practice and a daily average of 4.2 h (±2 h). The control group included 20 non‐musicians (nine female, 19 right‐handed, mean age 26.9 ± 5.3 years) without any prior formal or informal music training recruited from Danish Universities. Exclusion criteria were neurological, cardiorespiratory, mental disorders, chronic pain or pregnancy as well as frequent computer gamers (>9 h/week). The sample size was estimated based on previous publications using a similar approach (Biurrun Manresa et al., 2018; Fujioka et al., 2006; Mouraux et al., 2014) and to ensure 80% power for detecting a large effect size (Cohen's *d* ≥ 0.8) with an independent t‐test analysis at an alpha level of 0.05. All participants received written and verbal information about the scope of this study and provided written consent. The study was performed in accordance with the Declaration of Helsinki (General Assembly of the World Medical Association, 2013) and approved by the local ethics committee (Den Videnskabsetiske Komité for Region Nordjylland, N‐20170040).

### 2.2 Experimental procedure

At the beginning of the session, participants reported the demographic data and replied to a short interview about their musical practice. Afterwards, participants were seated in comfortable chairs and familiarized with the electrical test stimuli. To avoid excessive head and body movements, participants were instructed to fixate their gaze on a black cross (3 × 3 cm) displayed 1.5 m in front of them for the entire duration of each stimulation block.

The experiment consisted of two stimulation blocks with a sequence randomized and counterbalanced across participants. Each block comprised 30 stimuli belonging to one of two types of electrical stimulation to the right hand: (1) intra‐epidermal electrical stimulation, which predominantly activates Aδ nociceptors (Mouraux et al., 2010), and (2) low‐intensity transcutaneous electrical stimulation, which activates non‐nociceptive Aβ fibres (Burgess & Perl, 1967). To ensure that each stimulus was perceived and to maintain vigilance across time, participants had to press a button immediately after the perception of each stimulus (reaction time). Detection thresholds were recorded for each stimulation modality at baseline.

### 2.3 Acquisition of biographical data on musical practice

The accumulated training and daily practice were obtained by interviewing the musicians and asking them to retrospectively identify and self‐estimate the amount of practicing in different age periods (Bengtsson et al., 2005). The term ‘musical practice’ was defined as the time playing their instruments (i.e. classes, home training, rehearsals and concerts). First, it was asked when they first started practicing their instrument. Then, it was asked the average hours of musical practice per week in the different age periods. Those periods were (i) from the age of onset to age 7, (ii) from age 8–12, (iii) from age 13–16 and (iv) from age 17 to the time of the experiment. The total amount of accumulated training was calculated by summing the hours for the four different age periods. Finally, the current daily practice was obtained by asking the musicians how many hours they were training during the 7 days before the day of the experiment.

### 2.4 Electrical stimulation

To ensure that the stimuli remained selective for their respective fibres, the intensity was individually adjusted to twice the detection threshold (Mouraux et al., 2010). Both nociceptive and non‐nociceptive stimuli consisted of two rapidly succeeding constant‐current square‐wave pulses with a duration of 0.5 ms each, an inter‐pulse interval of 5 ms, and an inter‐stimulus interval that randomly varied between 8 and 10 s (Mouraux et al., 2014). The electrical stimuli were controlled using custom‐made software (‘Mr. Kick’, Aalborg University) and delivered by a constant‐current electrical stimulator (Digitimer DS5, Digitimer Ltd.).

Nociceptive stimuli were delivered using intra‐epidermal electrical stimulation (Inui et al., 2002). Stimuli were delivered using a stainless steel concentric bipolar needle electrode developed by Inui et al. (2002), consisting of a needle cathode (length: 0.1 mm, Ø: 0.2 mm) surrounded by a cylindrical anode (Ø: 1.4 mm). Gently pressing the device against the skin inserted the needle electrode into the epidermis of the dorsum of the right hand, which clearly elicited a burning/pricking sensation when stimulated. Given that low intensities are used, these stimuli predominantly activate nociceptive Aδ fibres (Mouraux et al., 2010).

Non‐nociceptive stimuli were elicited using low‐intensity transcutaneous electrical stimulation. Stimuli were delivered through a pair of digital ring electrodes (Digitimer Ltd.) and applied to the first two phalanges of the right index finger (1‐cm interelectrode distance). Given that low intensities are used, these stimuli predominantly activate non‐nociceptive Aβ fibres (Burgess & Perl, 1967).

### 2.5 Behavioural measures

Detection thresholds for nociceptive and non‐nociceptive stimuli were estimated using a staircase procedure (Mouraux et al., 2010). The initial stimulus intensity was 30 μA for the nociceptive and 100 μA for the non‐nociceptive stimulation, and the initial step sizes were 50 μA and 500 μA, respectively. After the first staircase reversal, the step size was reduced to 10 μA and 100 μA, respectively. The procedure was interrupted after the occurrence of three staircase reversals at the final step size. The detection thresholds were estimated by averaging the intensity of the stimuli at which these three reversals occurred.

The participants were instructed to push a button held in their left hand as soon as they perceived the stimulus. The mean reaction time (RT) across the 30 stimulations recorded relative to stimulus onset was extracted. RTs greater than 1000 ms were considered undetected.

### 2.6 Electrophysiological measures

Electroencephalographic (EEG) activity was recorded using an active electrode cap (g.SCARABEO, g.tec, Medical Engineering GMBH, Austria). The electrode montage included 64 electrodes according to the modified 10–20 system. During the recording, the EEG signals were amplified and digitized using a sampling rate of 1200 Hz and a left earlobe (A1) reference (g.Hlamp, g.tec, Medical Engineering GMBH). The ground electrode was placed at position AFz. The impedance of all electrodes was kept below 20 kΩ and assessed by the EEG system software (g.Recorder, g.tec, Medical Engineering GMBH).

Event‐related potentials (ERPs) were analysed offline using EEGLAB v.14.1.1(Delorme & Makeig, 2004) running under MATLAB (The Mathworks). Data were band‐pass filtered (0.5–40 Hz), followed by an Infomax independent component analysis using the in‐built EEGLAB function runica to identify and remove components associated with noise (e.g. eye movement, eye blinks, cardiac, muscle artefacts; Jung et al., 2000). Continuous data were segmented into 1.5 s epochs, stimulus‐locked from −500 to 1000 ms with time 0 corresponding to the stimulus onset. Baseline correction was made using the −500 to −10 ms window. For each subject and stimulus type, baseline‐corrected epochs were further averaged to extract the ERPs of interest (Kunde & Treede, 1993; Mouraux et al., 2010).

For the ERPs in response to nociceptive stimuli, N100, N200 and P300 components were analysed. The N100 component, commonly labelled in pain research as N1, was defined as the first major negative deflection occurring within the 60 ms time window preceding the N200 component (i.e. 100–160 ms), and measured with the recommended temporal–frontal montage (T7‐Fz; Valentini et al., 2012). The N200 and P300 components, labelled in pain research as N2 and P2, respectively (Cruccu et al., 2008), were identified with the recommended central‐earlobe montage (Cz‐A1). The N200 was defined as the first major negative deflection after stimulus onset, while P300 was defined as the first major positive deflection occurring after stimulus onset (Cruccu et al., 2008). For the ERPs in response to non‐nociceptive stimulation, the N140 (analogous to N200), P200 and P300 were determined using the midline Cz‐A1 montage (Shimojo et al., 2000).

For the non‐nociceptive stimulation, exploratory statistical analyses were performed on the following P50 and P100 components to assess the effects of extensive sensorimotor training. For the nociceptive stimulation, an exploratory analysis was performed on a positive peak at the latency of 200 ms (labelled P200) that is normally concealed in response to a nociceptive stimulus. These peak latencies were chosen on the basis of previous research (Miltner et al., 1989; Polich, 2007), and visual inspection.

In order to avoid a researcher‐biased ERP peak selection, ERP components were analysed using specific time windows of interest, which were centred at the peak latency of each ERP component and extended before and afterwards accordingly. The following time windows for non‐nociceptive stimulation were extracted: 45–55 ms (P50), 80–120 ms (P100), 100–220 ms (complex N140/P200) and 280–320 ms (P300). Similarly, the following time windows for nociceptive stimulation were extracted: 100–160 ms (N100), 100–220 ms (N200/P200) and 300–500 ms (P300).

**FIGURE 1:**
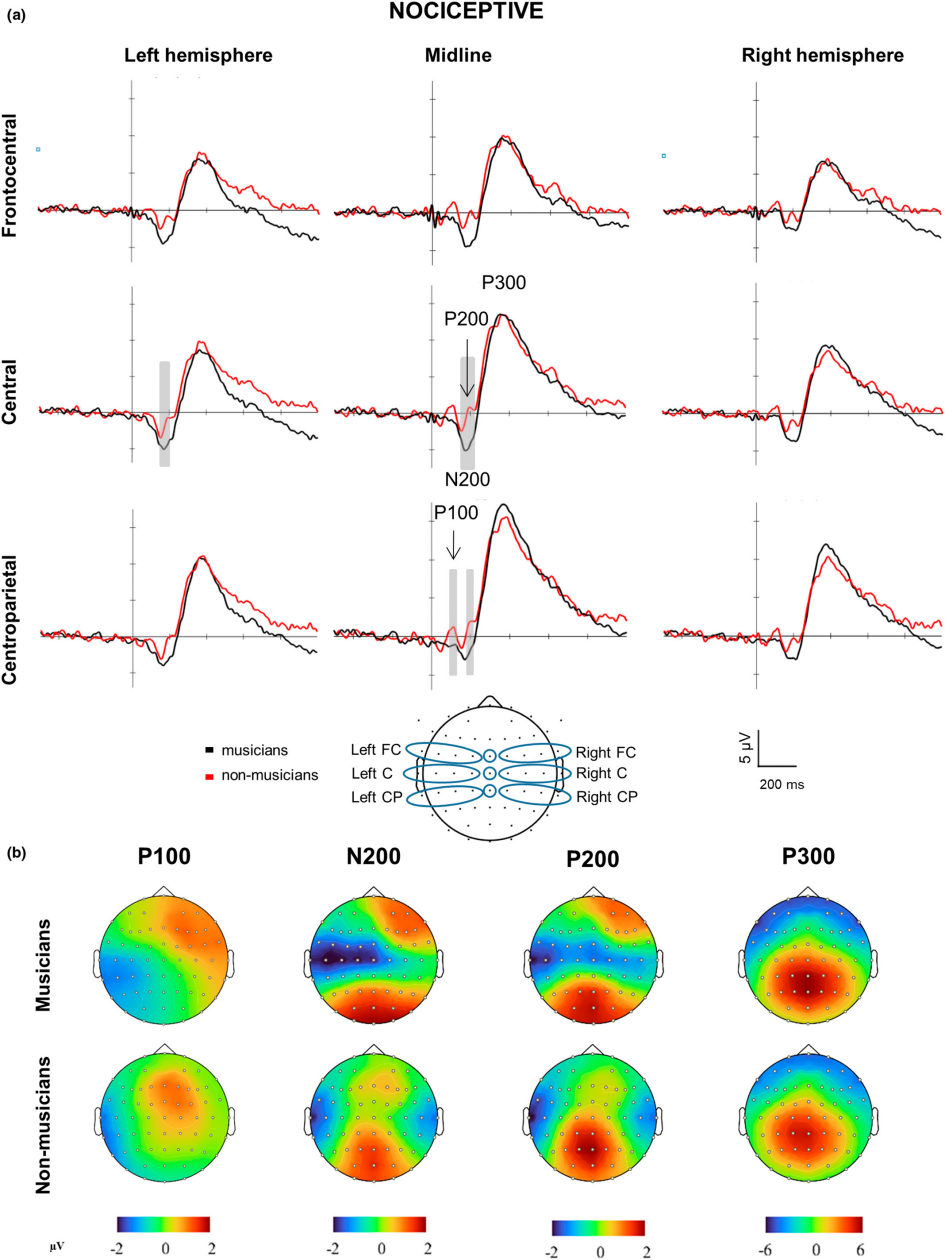
Brain responses and topographies of nociceptive stimuli. (a) Grand‐averaged event‐related potentials elicited by the nociceptive electrical stimulation at the hand and illustrated at nine topographical regions of interest (ROIs; bottom center) in musicians (red lines) and non‐musicians (blue lines). ROIs: frontocentral (FC), central (C) and centroparietal (CP). Marked time periods windows in grey indicate time periods and ROIs with significant differences between musicians and non‐musicians (*p* < 0.05). Negative is plotted downward. Amplitudes across all ROIs indicate larger N200 and smaller P100 and P200 components. (b) Amplitude scalp topography of each nociceptive component in musicians and non‐musicians. Scalp topographies shown are generated at 100 ms (P100), 180 ms (N200), 200 ms (P200), and 360 ms (P300).

**FIGURE 2:**
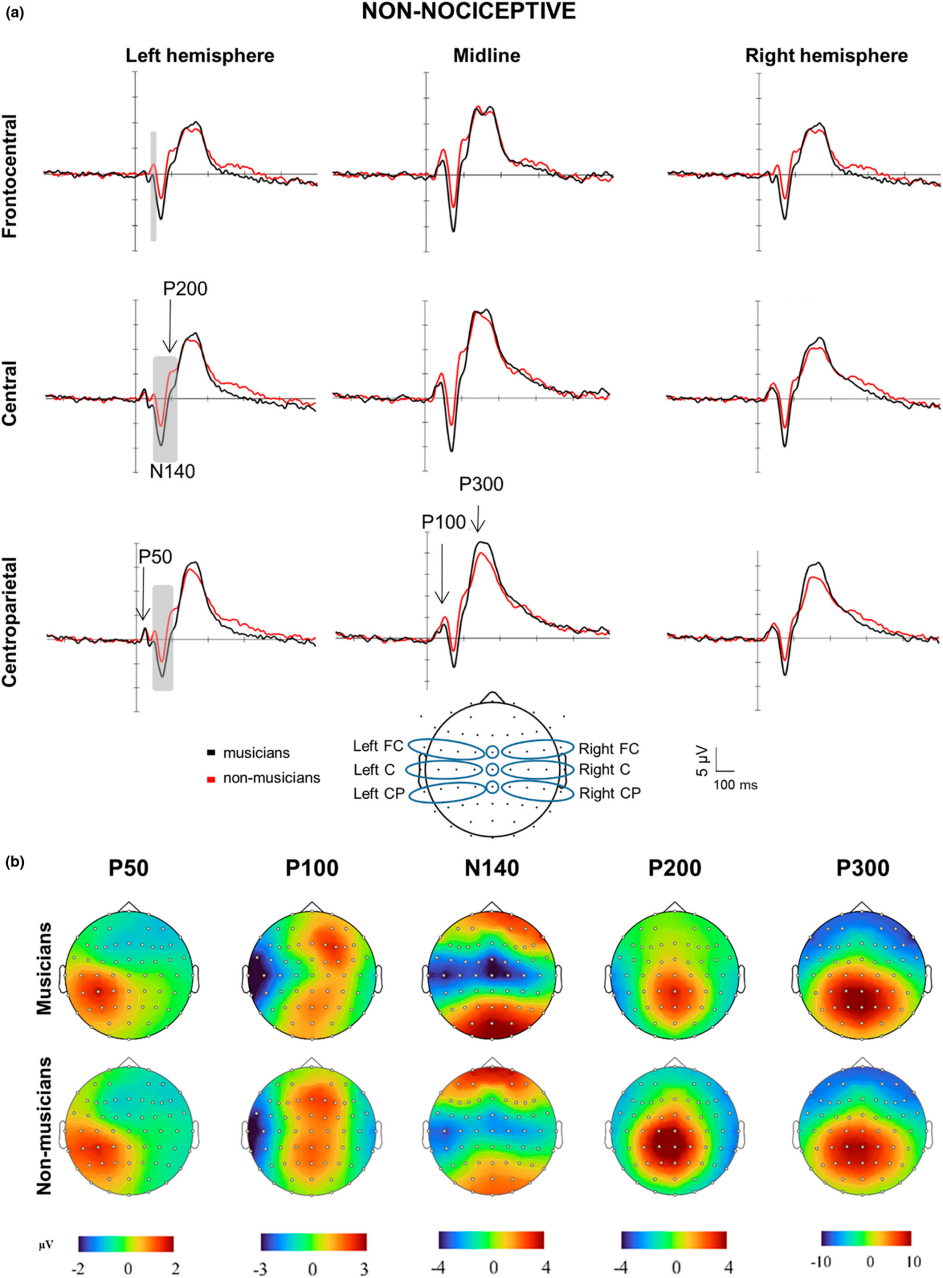
Brain responses and topographies of non‐nociceptive somatosensory stimuli. (a) Grand‐averaged event‐related potentials elicited by the non‐nociceptive electrical stimulation at the hand and illustrated at nine topographical regions of interest (ROIs; bottom centre) in musicians (red lines) and non‐musicians (blue lines). ROIs: frontocentral (FC), central (C) and centroparietal (CP). Marked time periods windows in grey indicate time periods and ROIs in which there were significant differences between musicians and non‐musicians (*p* < 0.05). Negative is plotted downward. Amplitudes across all ROIs were larger for the N140 and smaller for the P100 and P200 components in musicians compared to non‐musicians. (b) Amplitude scalp topography of each non‐nociceptive component in musicians and non‐musicians. Scalp topographies shown are generated at 45 ms (P50), 100 ms (P100), 140 ms (N140), 200 ms (P200) and 300 ms (P300).

ERP time windows were subsequently statistically evaluated within 9 topographical regions of interest (ROIs; see Figures 1 and 2): left frontocentral (FC1, FC3, FC5, TF7), right frontocentral (FC2, FC4, FC6, TF8), left central (C1, C3, C5, T7), right central (C2, C4, C6, T8), left centroparietal (CP1, CP3, CP5, TP7), right centroparietal (CP2, CP4, CP6, TP8) and the midline FCz, Cz and CPz electrodes. Latencies and amplitudes of each ERP component provided in Table 1 were extracted at their dominant scalp electrode.

### 2.7 ERPs source localization

Cortical source localization of nociceptive N200, P200 and P300 and non‐nociceptive N140, P200, and P300 were carried out using the Brainstorm toolbox (Tadel et al., 2011), a freely available software released under the GNU general public licence (http://neuroimage.usc.edu/brainstorm). The head model was computed using the default anatomy based on ICBM152, as no individual anatomy was available. The layout from the generic ICBM152 10–20 cap file was co‐registered with the default anatomy. The OpenMEEG toolbox (Gramfort et al., 2010), which consists of the symmetric Boundary Element Model (BEM), was used to calculate the EEG‐forward model. Individual noise covariance matrices were computed using single‐trial time windows before stimulus' onset (−500 to −10 ms). Unconstrained cortical sources were calculated at the single‐trial level by using the weighted minimum‐norm estimation (WMNE) approach and subsequently normalized with sLORETA (Pascual‐Marqui, 2002). The result was a three‐dimensional grid of 15,000 fixed dipoles. Single‐trial source data were averaged for each participant and across subjects to estimate active sources. Cortical source level activity is shown as absolute values with arbitrary units based on the normalization within the sLORETA algorithm.

### 2.8 Statistical analysis

Data are presented in text and table as means and standard deviations. Behavioural responses were statistically analysed with SPSS for Windows (IBM SPSS Statistics 26; IBM) and screened for assumptions of normality, sphericity and homogeneity using descriptive plots and the Shapiro–Wilk's, Mauchly's and Levene's statistical tests. Detection thresholds for nociceptive and non‐nociceptive stimuli were compared between groups using independent *t* tests. Reaction times were analysed using repeated measures analysis of variance (rmANOVA) with *Stimulation modality* (nociceptive or non‐nociceptive) as a repeated measures factor and *Group* (musicians and non‐musicians) as a between‐group factor. Significant factors or interactions were analysed post hoc using Bonferroni's procedure to correct for multiple comparisons.

ERPs a priori time windows and cortical sources were statistically analysed with Brainstorm. The entire ERP time window in response to nociceptive and non‐nociceptive stimuli was compared between groups using non‐parametric permutation tests repeated 1000 times. False discovery rate (FDR) correction of multiple comparisons was employed to control for Type I errors (Benjamini & Hochberg, 1995). For the cortical source, a priori time windows of 10 ms were analysed, centred at the peak latency of nociceptive N200, P200, P300 and non‐nociceptive N140, P200 and P300 ERP components. The whole‐brain activity was averaged across correspondent time windows and statistically compared between groups using a parametric one‐tailed power F test for unconstrained sources implemented in Brainstorm. FDR correction of multiple comparisons was employed to control for Type I errors.

Effect sizes for detection thresholds and ERPs were calculated using Cohen's *d* (*d* = 2 *t/*sqrt(df)) (Cohen, 2013). In addition, to control for a possible overestimation of the effect size due to the small sample size of the groups, Cohen's *d* was subsequently adjusted using the unbiased Cohen's *d*: *d*_unbiased_ *= d* [1 − (3/4 df − 1)] (Fritz et al., 2012). For the effect sizes of the reaction times, the eta‐squared (*η* ^2^, ratio of the effect variance to the total variance) values derived from the rmANOVA were reported along with the main effects of the *Stimulation modality* and *Group*. Correlations and linear regressions were computed to investigate whether the ERPs to non‐nociceptive and nociceptive stimulation could explain their respective stimulus detection thresholds and reaction times across all participants. In musicians, it was furthermore tested if accumulated musical training and daily practice time affected the amplitude and latency values of cortical ERPs, the reaction times and the stimulus detection thresholds. A subset of electrophysiological responses was selected based on a priori hypotheses and correlation matrices. Specifically, we selected the amplitudes and latencies registered at the respective dominant scalp electrode (Cz) of the main biphasic nociceptive N200/P300 (N2/P2) components and the non‐nociceptive N140/P200. The amplitudes and latencies that showed no significant correlations with the dependent variables (i.e. reaction time and stimulus intensity) were excluded from the model. Bootstrapping, a robust non‐parametric approach to hypothesis testing that does not make assumptions about the shape of the distribution of the variables (Efron & Tibshirani, 1985), was used to estimate the distribution of robust correlation and regression estimates (Salibian‐Barrera & Zamar, 2002). We used bias‐corrected and accelerated (BCa) 95% confidence intervals (CI) to test for significance, as they adjust for possible bias and skewness in the bootstrap distribution. If zero was not within the 95% confidence interval, we concluded that the indirect effect was significantly different from zero at *p* < 0.05, two‐tailed (Preacher & Hayes, 2004). For all tests used, the level of significance was set at *p* < 0.05.

## 3 RESULTS

### 3.1 Behavioural measures

#### 3.1.1 Stimulus characteristics

Nociceptive intra‐epidermal electrical stimulation induced a pricking sensation in all participants, except for one subject of the non‐musician group, who neither detected nor perceived the stimuli and was therefore excluded from the corresponding analysis. Non‐nociceptive electrical stimulation elicited a sensation of touch in all participants. Detection thresholds and reaction times confirmed that nociceptive and non‐nociceptive stimulation selectively activated their corresponding fibres. That is, according to the kind of fibres and the physical characteristics of each electrode (Poulsen et al., 2020), nociceptive stimulation required lower stimulus intensity and generated slower reaction times, in line with the characteristics of Aδ‐fibres, whereas non‐nociceptive stimulation required higher stimulus intensity and generated faster reaction times, in line with the characteristics of Aβ fibres.

#### 3.1.2 Stimulus detection thresholds

No significant differences in detection thresholds were found for nociceptive (*t*_31.08_ = 1.51, *p* = 0.136; *d*_unb_ = 0.52) and non‐nociceptive stimuli (*t*_38_ = −0.28, *p* = 0.777; *d*_unb_ = 0.09) between groups (Table1).

#### 3.1.3 Reaction times

Reaction time analysis (Table 1) revealed significant effects of *Stimulation modality* (*F*_1,37_ = 47.014, *p* < 0.001; *η* ^*2*^ = 0.56) and *Group* (*F*_1,37_ = 7.198, *p* = 0.011; *η* ^*2*^ = 0.16). Post hoc comparisons showed that reaction times were slower for the nociceptive than for the non‐nociceptive stimulation in both groups (*p* < 0.001), and that musicians responded faster than non‐musicians to both nociceptive and non‐nociceptive stimuli (*p* < 0.05). The interaction between *Stimulation modality* and *Group* was not significant (*F*_1,37_ = 0.73, *p* = 0.398; *η* ^*2*^ = 0.02).

**TABLE 1:** Mean (*±*SD) detection thresholds, detection rates and reaction times following nociceptive and non‐nociceptive electrical stimulation.

Intensity and reaction times	Musicians (n = 20)	Non‐musicians (n = 20)
Nociceptive detection threshold (μA)	153.1 ± 63.5	192.5 ± 95.6
Non‐nociceptive detection threshold (μA)	975.5 ± 316.6	948.5 ± 282.1
Nociceptive detection rate (%)	98 ± 5	92 ± 15
Non‐nociceptive detection rate (%)	100 ± 1	98 ± 4
Nociceptive reaction time (ms)	354 ± 55*‖	433 ± 139* ‖
Non‐nociceptive reaction time (ms)	265 ± 48*‖	315 ± 89* ‖

‖, Significantly different within kind of stimulations (*p* < 0.001); *, Significantly different between groups (*p* < 0.05).

### 3.2 Event‐related potentials in response to nociceptive stimulation

#### 3.2.1 N100

The N100 component was clearly identified in all non‐musicians and in 19 out of 20 musicians. Statistical analysis of N100 amplitudes across the entire time window, measured with the recommended temporal–frontal montage (T7‐Fz), revealed no significant group differences (*t*_36_ = 0.948; *p >* 0.5; *d*_unb_ = 0.30). Peak latencies of the N100 component revealed no significant group differences either (*t*_36_ = 0.012; *p* > 0.05; *d*_unb_ = 0.003).

#### 3.2.2 N200–P300

Nociceptive stimulation elicited a clear vertex potential constituted by a negative–positive biphasic wave (N200–P300 complex) in all participants with scalp dominance at the central midline electrode (Figure 1a,b, Table S1). Visual inspection indicated enlarged peak N200 amplitudes (Figure 1a) in musicians compared to non‐musicians. In addition, visual inspection of the N200 component time window also indicated a prominent positive component at 200 ms (labelled P200) in non‐musicians but not in musicians.

Statistical analysis of N200 amplitudes across the entire time window (Figure 1) showed larger amplitudes in musicians compared to non‐musicians at the left central (*t*_36_ = 3.2, *p* < 0.05; *d*_unb_ = 1.03), midline central (*t*_37_ = 3.5, *p* < 0.05; *d*_unb_ = 1.12) and midline centroparietal ROIs (*t*_37_ = 3.1, *p* < 0.05; *d*_unb_ = 0.9). Moreover, nociceptive P200 amplitude was smaller in musicians compared to non‐musicians at the left and midline central (*t*_37_ = 2.9, *p* < 0.05; *d*_unb_ = 0.94 and *t*_36_ = 3.7, *p* < 0.05; *d*_unb_ = 1.2, respectively) and at the centroparietal midline ROIs (*t*_37_ = 2.7, *p* < 0.05; *d*_unb_ = 0.93; Figure 1a).

Statistical analysis of P300 amplitudes across the entire time window revealed no significant group differences across ROIs (all *t*_37_ < 1.5; *p* > 0.63; *d*_unb_ < 0.48; Figure 1). Peak latencies of the N200, P200 and P300 components extracted at their dominant scalp electrodes (N200 and P200, Cz; P300 CPz; Table S1) revealed no significant group differences at their dominant scalp electrodes (all *t*_37_ < 0.73; *p* > 0.32; *d*_unb_ = 0.23).

Visual inspection of peak amplitudes also indicated a prominent component at 100 ms (labelled P100) at dominant frontal and central midline scalp distributions in the non‐musicians group (Figure 1a,b). The P100 amplitude across the complete time window was significantly smaller in musicians compared to non‐musicians at the midline centroparietal ROI (*t*_37_ = 3.0, *p* < 0.05; *d*_unb_ = 0.96). Peak latencies of the P100 component revealed no significant group differences (*t*_37_ = −0.483, *p* = 0.63; *d*_unb_ = 0.15).

### 3.3 Event‐related potentials in response to non‐nociceptive stimulation

All non‐nociceptive ERP components were clearly identified (Figure 2; Table S2) in all participants except for one non‐musician, in whom the EEG recording failed.

#### 3.3.1 N140–P200

The N140–P200 complex exhibited a clear negative–positive biphasic wave with a maximum scalp dominance at left (contralateral) central and midline Cz electrodes (Figure 2a,b). Visual inspection of peak amplitudes across all ROIs indicated a general enlargement of N140 and a reduction for the P200 component in musicians compared to non‐musicians.

Statistical analysis of N140–P200 across the entire time window showed significantly larger amplitudes for the N140 and smaller amplitudes for the P200 component in musicians compared to non‐musicians at the contralateral (left) central (N140: *t*_37_ = 2.9, *p <* 0.05; *d*_unb_ = 0.93; P200: *t*_36_ = 2.7, *p <* 0.05; *d*_unb_ = 0.87) and centroparietal ROIs (N140: *t*_37_ = 2.8, *p <* 0.05; *d*_unb_ = 0.90; P200: *t*_36_ = 3.2, *p <* 0.05; *d*_unb_ = 1.03; Figure 2a).

Peak latencies extracted at Cz showed no significant group differences for the N140 or the P200 components at their dominant scalp electrodes (N140: *t*_37_ = 0.08, *p* = 0.93; *d*_unb_ = 0.02; and P200: *t*_36_ = −1.05, *p* = 0.30; *d*_unb_ = 0.33).

#### 3.3.2 P300

The non‐nociceptive P300 component exhibited a prominent positive peak at 300 ms with a maximum scalp dominance at the centroparietal and parietal (Figure 2a,b).

Statistical analysis of the P300 time window showed no significant group differences across the nine ROIs (all *t*_37_ < 1.44; *p* > 0.15; *d*_unb_ < 0.47; Figure 2a,b). Peak latencies of the P300 extracted at CPz‐A1 showed no significant group differences (all *t*_37_ < 0.49; *p* > 0.62; *d*_unb_ < 0.16).

#### 3.3.3 P50 and P100 exploratory analyses

The exploratory analysis for P50 showed a left centroparietal‐dominant scalp distribution contralateral to the stimulation side (45–55 ms after stimulus onset). The P100 peak amplitude scalp distribution (90–110 ms after stimulus onset) was frontocentral and centroparietal.

Statistical analysis of the P50 time window showed no significant group differences across the nine ROIs (all *t*_37_ < 1.44; *p* > 0.15; *d*_unb_ < 0.46; Figure 2a,b). However, peak amplitudes for the P100 time window yielded a significantly smaller P100 amplitude in musicians compared to non‐musicians for the left frontocentral ROI (*t*_37_ = 3.1; *p* < 0.05; *d*_unb_ = 0.99; Figure 2a).

Peak latencies of the P50 extracted at CP3‐A1, as well as P100 extracted at CPz‐A1 showed no significant group differences (all *t*_37_ < 0.48; *p* > 0.53; *d*_unb_ < 0.15; Table 1).

### 3.4 Source maps in response to nociceptive stimulation

**FIGURE 3:**
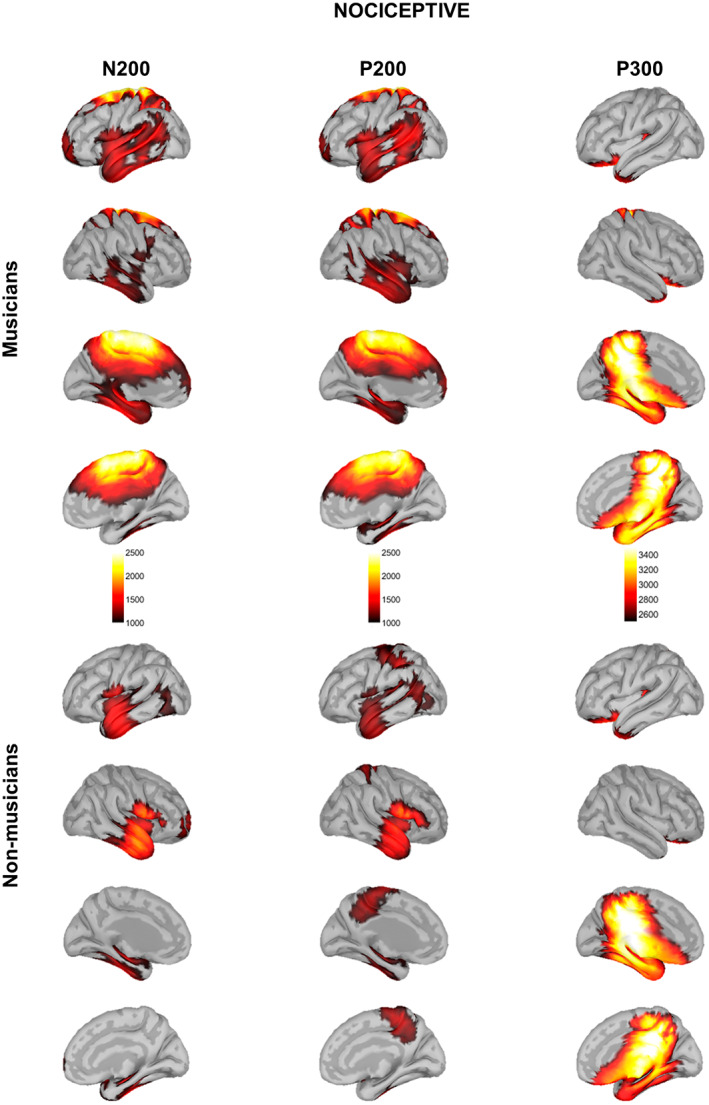
Source localization maps in response to nociceptive electrical stimulation in musicians (top) and non‐musicians (bottom). Source maps shown are generated at 180 ms (N200), 200 ms (P200) and 350 ms (P300).

For the nociceptive electrical stimulation, the source analysis revealed distinct activations for each ERP component (Figure 3).

#### 3.4.1 N200

In musicians, source reconstruction of N200 involved the left insula and its adjacent operculum; the left temporal pole, the left entorthinal and parahippocampal cortices; the bilateral superior, middle, and inferior temporal gyri (STG, MTG, and ITG, respectively); the bilateral anterior middle and posterior cingulate cortices (aMCC and PCC, respectively); the bilateral isthmus of the cingulate cortex; the bilateral precuneus; the bilateral paracentral lobule; the bilateral primary somatosensory and motor cortices (S1 and M1, respectively); the bilateral supplementary motor area (SMA), the superior frontal gyrus (SFG), and the left middle frontal gyrus (MFG).

Non‐musicians showed activation in the bilateral insula and its adjacent operculum; the bilateral STG, MTG, and ITG; the bilateral fusiform gyrus; the right entorthinal cortex; and the right prefrontal cortex.

**FIGURE 4:**
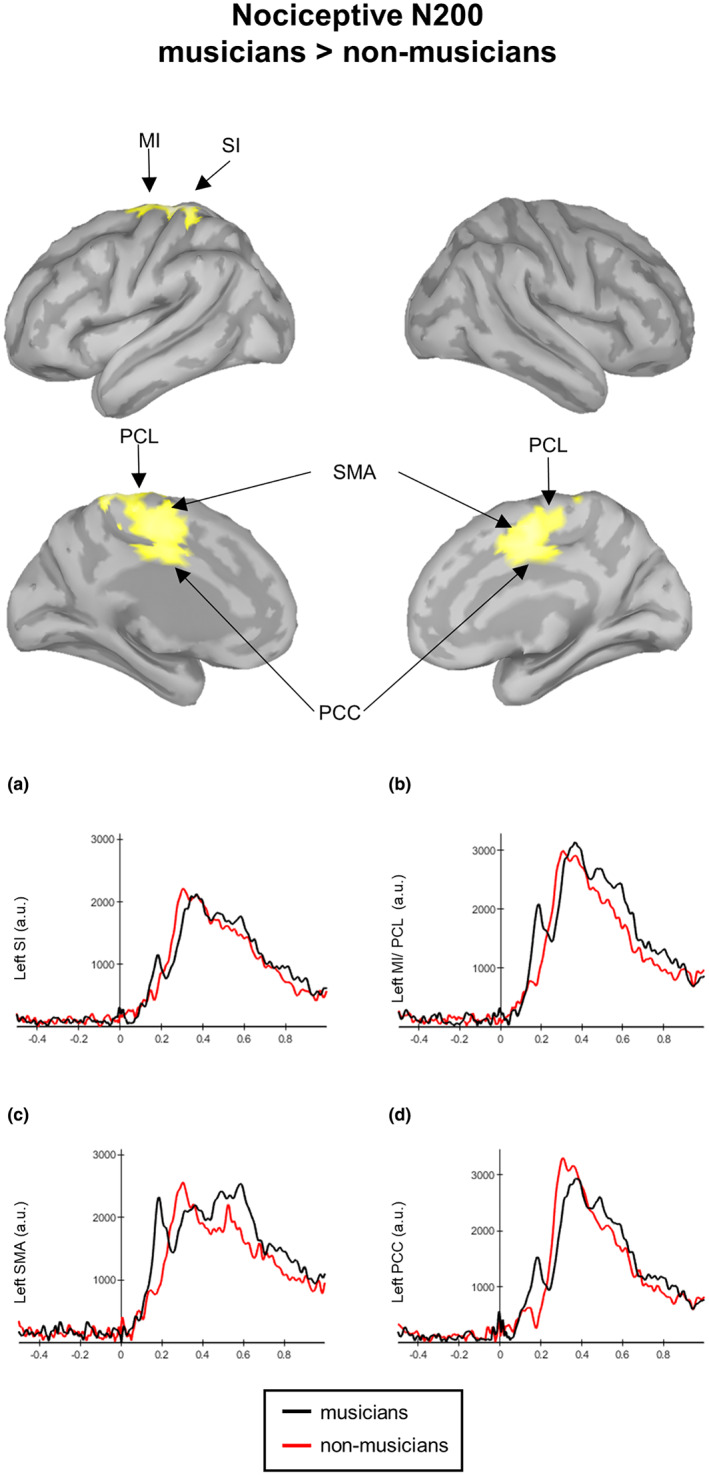
Contrast maps of whole‐brain activity between musicians and non‐musicians (red lines at N200 in response to nociceptive electrical stimulation. Significance thresholds were set at *p* < 0.05, and multiple comparisons were false discovery rate (FDR) corrected. Waveforms correspond to the entire time courses of the neural sources in musicians (black lines) and non‐musicians (red‐lines) at the left primary somatosensory cortex (SI, a), the left MI and paracentral lobule (MI/PCL, b), the left supplementary motor area (SMA, c) and the left posterior cingulate cortex (PCC, d).

Statistical analysis of whole‐cortical activity for N200 ERP (Figure 4, Table 2) revealed higher cortical activity in musicians compared to non‐musicians in the left primary somatosensory and motor cortices (SI and MI, respectively), the bilateral PCC, the bilateral paracentral lobule, and the bilateral SMA.

**TABLE 2:** MNI coordinates and local maxima of whole‐cortical differences (t‐contrasts) for nociceptive N200 and non‐nociceptive N140 cortical sources. T values of significantly activated peak voxels refer to MNI coordinates of regions with higher activation in musicians compared to non‐musicians. Only results that survived a false discovery rate (FDR) correction are shown. Labelling was performed using the Desikan–Killiany and Brodmann atlases implemented in Brainstorm.

Region		Coordinates MNI			*t*‐value	*d*_unb_
		*x*	*y*	*z*		
Nociceptive N200						
SI	L	−11	−33	77	2.80	0.90
MI	L	−10	−28	79	2.61	0.84
PCC	L	−5	−11	44	2.47	0.80
PCC	R	3	−7	44	2.48	0.80
PCL	L	−6	−26	57	2.51	0.81
PCL	R	4	−15	52	2.35	0.76
SMA	L	−1	−8	52	2.46	0.79
SMA	R	1	9	50	2.49	0.80
Non‐nociceptive N140						
Ant. Ins.	R	38	7	−15	1.92	0.62
OFC	R	45	43	−21	2.53	0.81
TP	R	29	24	−41	1.91	0.62
STG	R	52	17	−27	2.29	0.74
MTG	R	65	−11	−27	2.82	0.91
ITG	R	58	−9	−38	2.69	0.87
AG	R	53	−59	46	2.72	0.88
SMG	R	61	−44	49	2.43	0.78
SMA	L	−8	13	72	2.52	0.81
SMA	R	7	9	74	2.55	0.82
PCL	L	−2	−32	74	2.28	0.73
PCL	R	2	−12	65	2.06	0.66
PCC	L	−1	−36	46	2.00	0.64
PCC	R	1	−24	45	1.92	0.62
Prec	L	−1	−73	47	2.41	0.78
Prec	R	3	−75	39	2.44	0.79
SPL	L	−19	−88	44	2.46	0.79
SPL	R	27	−91	32	2.29	0.74

Abbreviations: AG, angular gyrus; ant. Ins., anterior insula; ITG, inferior temporal gyrus; L, left; MI, primary motor cortex; MNI, Montreal neurological institute; MTG, middle temporal gyrus; OFC, orbitofrontal cortex; PCC, posterior cingulate cortex; PCL, paracentral lobule; Prec., precuneus; R, right; SI, primary somatosensory cortex; SMA, supplementary motor area; SMG, supramarginal gyrus; SPL, superior parietal lobule; STG, superior temporal gyrus; TP, temporal pole.

#### 3.4.2 P200

Musicians displayed the activation of the bilateral insula and their adjacent operculum; the bilateral temporal pole and their adjacent STG, MTG and ITG; the bilateral fusiform gyrus; the left entorthinal and parahippocampal cortices; and the left angular gyrus. Moreover, musicians also showed activation in the bilateral aMCC, PCC, and the bilateral isthmus of the cingulate cortex; the bilateral precuneus; the bilateral paracentral lobule; the bilateral SMA; the bilateral SFG; and the left MFG.

Non‐musicians showed activation in the bilateral insula and its adjacent operculum; the bilateral temporal pole and their adjacent STG, MTG, and ITG; the left fusiform gyrus; the left angular gyrus; the bilateral paracentral lobule; the left SI and MI; as well as the bilateral precuneus.

Statistical analysis of whole‐cortical activity for P200 ERP yielded no significant differences.

#### 3.4.3 P300

Musicians and non‐musicians displayed brain activation in the posterior left insula cortex; the bilateral orbitofrontal cortex (OFC); the bilateral PCC and the bilateral isthmus of the cingulate cortex; the bilateral parahippocampal and entorthinal cortices; the bilateral fusiform gyrus; the bilateral temporal pole; the bilateral paracentral lobule; and the bilateral precuneus.

Statistical analysis of whole‐cortical activity for P300 ERP yielded no significant differences.

### 3.5 Source maps in response to non‐nociceptive stimulation

**FIGURE 5:**
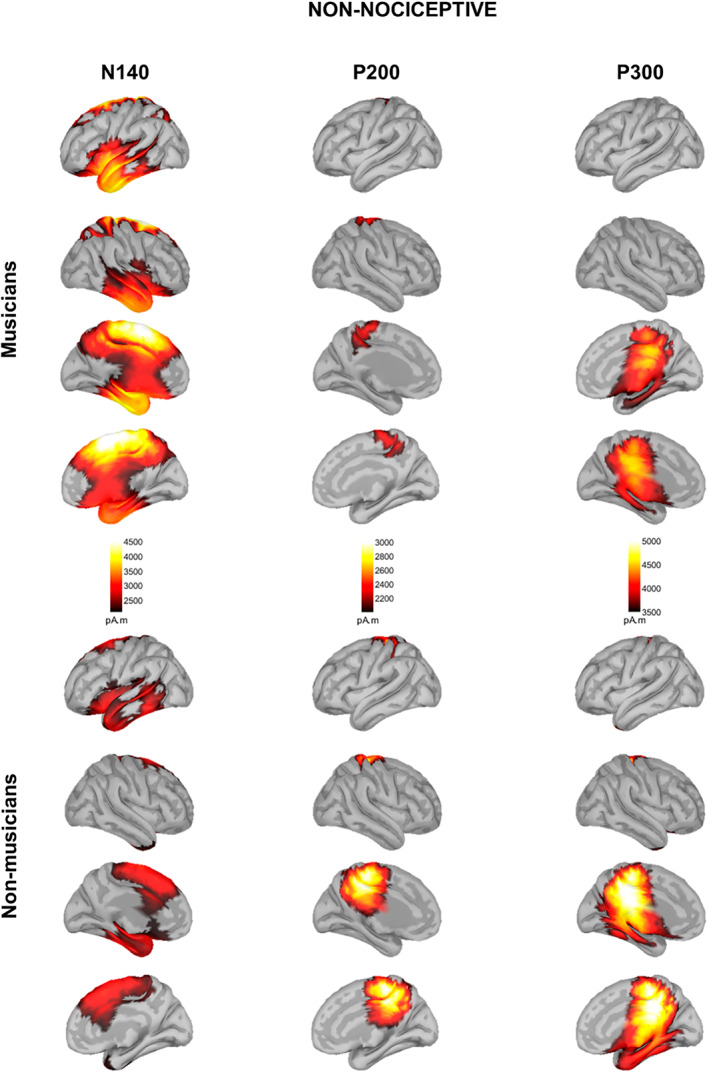
Source localization maps in response to non‐nociceptive electrical stimulation in musicians (top) and non‐musicians (bottom). Source maps shown are generated at 140 ms (N140), 200 ms (P200) and 300 ms (P300).

For the nociceptive electrical stimulation, the source analysis revealed distinct activations for each ERP component (Figure 5).

#### 3.5.1 N140

Musicians displayed bilateral activation of the anterior insula and their adjacent operculum; the OFC; the temporal pole, the entorthinal and the left parahippocampal cortices; the bilateral STG, MTG and ITG; the bilateral aMCC, the bilateral PCC; the bilateral precuneus; the bilateral paracentral lobule; and the bilateral SFG.

Non‐musicians showed activation in the left anterior insula and its adjacent OFC; the left temporal pole; the left STG and ITG; the left fusiform gyrus; the right aMCC; and the bilateral SFG.

**FIGURE 6:**
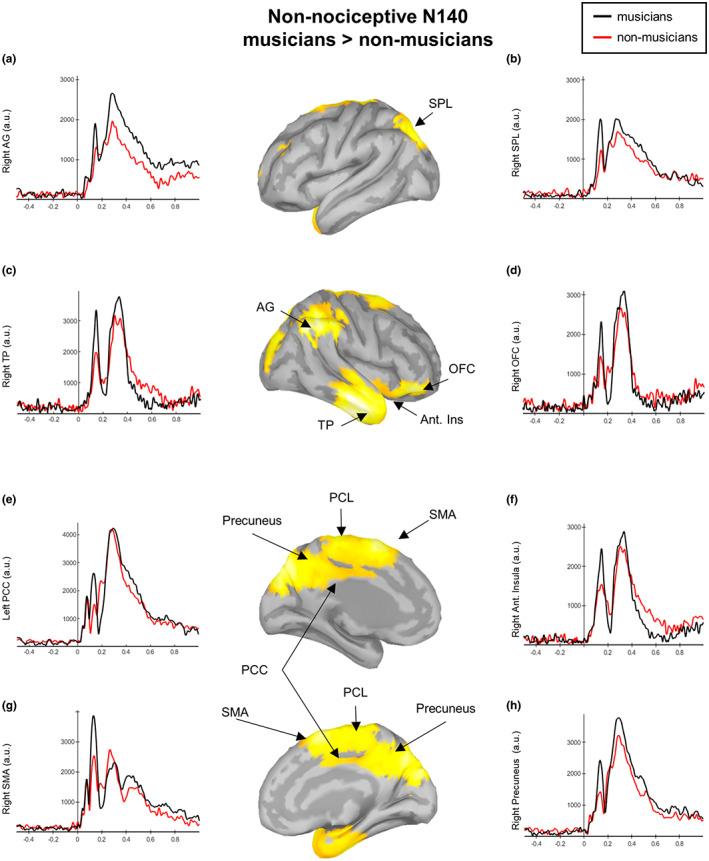
Contrast maps of whole‐brain activity between musicians and non‐musicians at N140 in response to non‐nociceptive electrical stimulation. Significance thresholds were set at *p* < 0.05, and multiple comparisons were false discovery rate (FDR) corrected. Waveforms correspond to the entire time courses of the neural sources in musicians (black lines) and nonmusicians (red lines) at the right angular gyrus (AG, a), the right superior parietal lobule (SPL, b), the right temporal pole (TP, c), the right orbitofrontal cortex (OFC, d), the left posterior cingulate cortex (PCC, e), the right anterior insula (f), right supplementary motor area (SMA, g) and the right precuneus (h).

Statistical analysis of whole‐cortical activity for N140 ERP revealed higher cortical activity in musicians compared to non‐musicians (Figure 6, Table 2). In particular, in the right anterior insula and adjacent OFC; the right temporal pole and the right STG, MTG and ITG; the right angular and supramarginal gyrus; the bilateral SMA; the bilateral paracentral lobule; the bilateral PCC; the bilateral precuneus; and the bilateral superior parietal lobule (SPL).

#### 3.5.2 P200

Musicians displayed activation in the bilateral paracentral lobule and the bilateral precuneus. Non‐musicians showed bilateral activation in the paracentral lobule, the precuneus, the PCC and the isthmus of the cingulate cortex.

Statistical analysis of whole‐cortical activity for non‐nociceptive P200 ERP yielded no significant differences.

#### 3.5.3 P300

Musicians and non‐musicians displayed the activation of the bilateral PCC and the bilateral isthmus of the cingulate cortex; the bilateral paracentral lobule; the bilateral precuneus and the bilateral parahippocampal cortex. Musicians, moreover, showed the activation of the right entorthinal cortex and the right fusiform gyrus.

Statistical analysis of whole‐cortical activity for non‐nociceptive P300 ERP yielded no significant differences.

### 3.6 Correlations and regressions between ERPs components and behavioural measures

#### 3.6.1 Across participants, non‐nociceptive N140 and nociceptive N200 components correlate with reaction times

**FIGURE 7:**
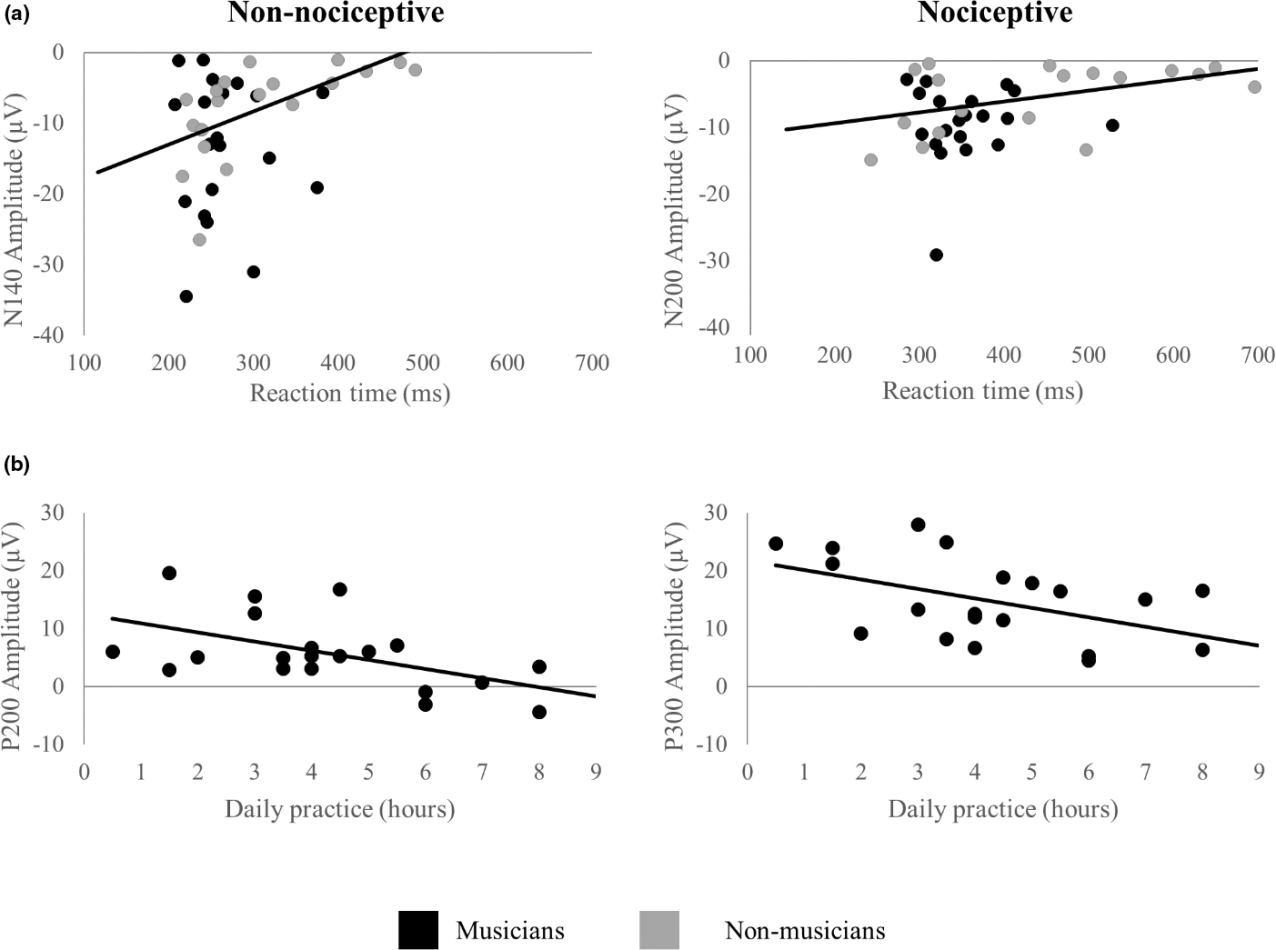
Significant correlations of event‐related potentials, reaction times and daily practice. (a) The non‐nociceptive N140 (left) and nociceptive N200 (right) peak amplitudes correlate with their respective non‐nociceptive and nociceptive electrical stimulation reaction times. Musicians are represented by black dots and non‐musicians by grey dots. (b) In musicians, the amount of daily practice (h) correlates with the peak amplitudes of the non‐nociceptive P200 and the nociceptive P300 components. Fit lines indicate correlations between respective variables.

Reaction times showed a positive significant correlation with their respective non‐nociceptive N140 (*r* = 0.49, *p* = 0.002, *BCa CI* = 0.29 to 0.65) and nociceptive N200 (*r* = 0.35, *p* = 0.029, *BCa CI* = 0.12 to 0.57) peak amplitudes (Figure 7a), but not with their respective latencies (*p* > 0.05). Reaction times did not significantly correlate with the non‐nociceptive P200 and nociceptive P300 latencies and amplitudes (all *p* > 0.05).

#### 3.6.2 Across participants, the non‐nociceptive P200 and nociceptive P300 correlate with stimulus detection thresholds

Stimulus detection thresholds for the non‐nociceptive stimulus showed a negative significant correlation with the magnitude of the corresponding P200 peak amplitudes (*r* = −0.40, *p* = 0.010, *BCa CI* = −0.57 to −0.15). Non‐nociceptive detection thresholds were neither significantly correlated with the P200 latency nor with the N140 amplitude and latency (all *p* > 0.05).

Stimulus detection thresholds for the nociceptive stimulus showed a negative significant correlation with the latencies of the corresponding P300 peak (*r* = −0.43, *p* = 0.006, *BCa CI* = −0.66 to −0.26). Nociceptive detection thresholds were not significantly related to the respective nociceptive N200/P300 amplitudes nor to the latencies of the N200 (all *p* > 0.05).

#### 3.6.3 The amount of daily sensorimotor practice in musicians correlates with non‐nociceptive and nociceptive components as well as reaction times

In musicians, the amount of daily practice showed a negative significant correlation with the magnitude of the non‐nociceptive P200 (*r* = −0.53, *p* = 0.016, *BCa CI* = −0.75 to −0.30) and the nociceptive P300 (*r* = −0.45, *p* = 0.048, *BCa CI* = −0.66 to −0.13) amplitudes (Figure 7b) as well as with the non‐nociceptive (*r* = −0.47, *p* = 0.035, *BCa CI* = −0.77 to −0.06) and nociceptive (*r* = −0.49, *p* = 0.027, *BCa CI* = −0.73 to −0.20) reaction times. However, when extreme cases are removed, the correlations between daily practices and reaction times lacked statistical significance (nociceptive: *r* = −0.41, *p* = 0.08, *BCa CI* = −0.69 to −0.94; non‐nociceptive: *r* = −0.20, *p* = 0.41, *BCa CI* = −0.46 to 0.02).

In order to test the relationship between daily practice and the magnitude of the evoked response amplitudes, we included musicians in a regression, using stimulus intensity and daily practice as predictors. Higher non‐nociceptive detection thresholds explained smaller P200 amplitudes, accounting for 22.7% of the variance (*F*_1,19_ = 5.29, *R* ^2^ = 0.23, *p* = 0.034, *BCa CI* = −0.02 to −0.01). By adding daily musical practice, the model explained 43.8% of the variance (*F*_2,19_ = 6.61, *R* ^2^ = 0.44, *p* = 0.008, *BCa CI* = −3.05 to −0.72). Thus, more daily practice significantly improved the prediction of smaller P200 amplitudes (*F*_change (1,19)_ = 6.36; *R* ^2^_change_ = 0.21, *p* = 0.022), accounting for an additional 21.1% of the variance.

## 4 DISCUSSION

Using musicians as a model for use‐dependent plasticity, the present study investigated whether prolonged and repeated sensorimotor training may alter the neural mechanisms of pain processing in healthy individuals. Results showed that, in response to similar stimulus intensities, healthy musicians showed larger non‐nociceptive N140 and nociceptive N200 peak amplitudes, smaller P200 peak amplitudes in response to both nociceptive and non‐nociceptive stimulation and displayed faster RTs. Notably, daily sensorimotor training in musicians was associated with non‐nociceptive P200, and nociceptive P300 amplitudes, emphasizing the use‐dependent nature of this modulation. Moreover, larger non‐nociceptive N140 and nociceptive N200 components were associated with faster RTs across all participants. This novel evidence provides first direct support for a putative model, suggesting that the same mechanisms by which repetitive sensorimotor training and multimodal integration can enhance selectivity to non‐nociceptive stimuli may also facilitate neural responses to nociceptive cues in healthy humans.

### 4.1 Effects of extensive sensorimotor training on non‐nociceptive processing

In the present study, extensive sensorimotor training facilitated upstream perceptual information processing and top‐down response control (reaction time) during non‐nociceptive electrical stimulation, which was used to assess the effects of sensorimotor training on the dorsal column–lemniscal pathway, as indicated by enlarged N140 and decreased P200 amplitudes. Across all participants, the N140 amplitude was associated with the reaction times to stimulus detection, analogous to previous reports (Talsma et al., 2007), whereas in musicians, the hours of daily musical training explained the decreased P200 amplitudes. Moreover, source analysis of N140 showed higher activation of areas involved in salience/sensory detection and sensorimotor processing (i.e. right anterior insula and adjacent OFC; the right temporal pole and adjacent STG, MTG and ITG; the right angular and supramarginal gyrus as well as the bilateral SMA, the PCL, the PCC, the precuneus and the SPL) in musicians compared to non‐musicians. This result is supported by previous resting‐state fMRI studies, in which musicians compared to non‐musicians showed an increased temporal correlation in blood oxygenation level‐dependent (BOLD) signals between the insular cortex and the cingulate, orbitofrontal, dorsolateral prefrontal cortices as well as the right angular and supramarginal gyri (Zamorano et al., 2017). Considering that sensorimotor experience in musicians has been linked to enhanced multimodal perception and correspondingly faster motor responses (Landry & Champoux, 2017), the current results suggest that the N140/P200 complex may reflect the electrophysiological correlate of this superior behavioural performance.

In the general population, enhanced N140 components have been reported in response to multisensory (tactile and visual) relative to unisensory stimulation (Eimer & Driver, 2000; Ohara et al., 2006), supporting the theory that multimodal integration facilitates a more robust sensory perception that shapes cross‐modal effects on evoked neural responses (Driver & Noesselt, 2008; Talsma et al., 2010). Musical training has long been known to enhance auditory evoked potentials such as the N100 (Pantev et al., 1998; Shahin et al., 2004), a negative peak recorded between 100 and 200 ms that is equivalent to the tactile N140 and the nociceptive N200. Similar effects have been reported in non‐musicians after 1 year of musical training (Fujioka et al., 2006), indicating that the temporal synchrony of neurons may be augmented through musical experience. Also, the early somatosensory cortical responses are enhanced in trumpet players compared to non‐musicians when tactile (lip stimulation) and auditory (trumpet tones) cues are presented simultaneously (Schulz et al., 2003), thus providing evidence of cross‐modal reorganization associated with multimodal sensorimotor training. The nature of this cortical facilitation is moreover task‐specific, as enhanced N100 potentials in musicians have been linked to the timbre of their principal instrument (Pantev et al., 2001).

Skilled performance requires the precisely timed integration, segregation and prediction of ongoing auditory, visual, tactile, proprioceptive and visceral feedback (Kleber et al., 2013; Lee & Noppeney, 2011; Schirmer‐Mokwa et al., 2015), which has also been associated with neural adaptations related to enhanced (insula‐based) salience detection and attentional selectivity, as well as increased functional connectivity between the insula, cingulate and somatosensory cortices to facilitate the access to the motor system (Kleber et al., 2017; Luo et al., 2014; Schirmer‐Mokwa et al., 2015; Zamorano et al., 2017). Taking this evidence into account and the higher cortical activation of salience and sensorimotor areas in musicians compared to non‐musicians in the current study, the present data suggest that extensive sensorimotor training and corresponding multisensory integration may specifically facilitate the priming of neural responses in brain areas where multimodal stimuli converge (Lu et al., 2014; Murray et al., 2005; Paraskevopoulos et al., 2012).

### 4.2 Effects of extensive sensorimotor training on nociceptive processing

Larger N200 amplitudes were found in the current study during nociceptive stimulation in healthy musicians compared to healthy non‐musicians. Moreover, prior use‐dependent plasticity influenced the appearance of an evoked cortical activity around 200 ms (similar to the non‐nociceptive P200) in non‐musicians, which is normally concealed in response to nociceptive stimulus (Miltner et al., 1989). Results also showed that the individual N200 amplitudes across all participants were associated with the reaction times to nociceptive stimulation, where reaction times were generally faster in musicians. Source analysis of ERP components in both groups indicated that the N200‐P300 components are predominantly generated in the insula, its adjacent operculum and the cingulate cortex, coinciding with prior literature (Garcia‐Larrea et al., 2003). However, statistical comparisons indicated that nociceptive N200 was also linked to the activation of sensorimotor regions (i.e. left SI and MI, the bilateral PCC, the bilateral paracentral lobule and the bilateral SMA) and that the pattern of activation was reversed in musicians compared to non‐musicians. This opposite activation of cortical activity in the SMA, cingulate cortex and PCL is in line with previous resting‐state fMRI studies in which musicians with persistent pain showed a reversed pattern of spontaneous BOLD activity compared to chronic pain patients with no experience performing extensive repetitive tasks (Zamorano et al., 2019). This suggests that extensive sensorimotor training may trigger adaptations in neural systems that overlap with pain processing.

Increased electrophysiological activity in nociceptive ERP components has also been observed in experimental pain models of secondary hyperalgesia. These studies showed that short periods of sustained nociceptive input delivered by high‐frequency nociceptive stimulation (HFS) on the skin not only induce hypersensitivity and faster reaction times, but also enhance the N200 components elicited by activation of Aδ‐ and C‐fibre nociceptors around the stimulated area (Biurrun Manresa et al., 2018; Lenoir et al., 2018). The underlying mechanisms following sustained nociceptive HFS have been associated with long‐term potentiation (LTP) of excitatory synaptic transmission, a key feature for improving signal processing and sensory transmission (Froemke et al., 2013), between peripheral nociceptive fibres within dorsal horn neurons projecting to the parabrachial area in the brainstem (Ikeda et al., 2003). In addition, sustained nociceptive HFS can also enhance the non‐nociceptive evoked N100 peak amplitude (the negative peak recorded between 100 and 200 ms and equivalent to N140 and N200) in response to vibrotactile (van den Broeke & Mouraux, 2014) and visual stimulation (Torta et al., 2017), suggesting that sustained nociceptive stimulation may also engage LTP mechanisms in supra‐spinal multisensory areas, such as the insula and ACC (Zhuo, 2014).

Sustained high‐frequency non‐nociceptive stimulation (i.e. transcutaneous electrical nerve stimulation, TENS), on the other hand, diminishes pain perception, leads to hypoalgesia and induces a reduction of the N100, N200 and P300 amplitudes to nociceptive stimuli (Peng et al., 2019), contrary to the effects of extensive sensorimotor training on nociceptive pathways reported in the current study. An explanation for the different findings between experimental (TENS) and an ecological (i.e. musical training) model of non‐nociceptive stimulation may be a difference in their underlying neural mechanisms. That is, experimental non‐nociceptive TENS is a passive unisensory stimulation performed for only 20 min (Sluka & Walsh, 2003), activating Aβ fibres and inhibiting incoming nociceptive inputs transmitted via Aδ and C fibres at the spinal level with the contribution of a supra‐spinal descending inhibitory mechanism (Peng et al., 2019). Musical training, in contrast, represents long‐term (i.e. years of) active multisensory stimulation, which is known to facilitate supra‐spinal LTP‐like mechanisms (Bütefisch et al., 2000; Zatorre et al., 2012) that may be similar to the mechanisms of nociceptive HFS. Moreover, as mentioned above, musical training enhances the precisely timed integration, segregation and prediction of multisensory cues (Kleber et al., 2013; Lee & Noppeney, 2011; Schirmer‐Mokwa et al., 2015) to enhance salience detection and attentional selectivity. Thus, it is possible that these training‐related neural adaptations may also shape the salient detection and integration of nociceptive cues.

Altogether, it is likely that LTP‐like mechanisms associated with extensive sensorimotor training and multisensory integration may not only facilitate the transmission of task‐specific non‐nociceptive sensory inputs, but possibly also enhance multisensory signal processing at the spinal and supra‐spinal pathways, which prime the perceptual processing of nociceptive signals. This explanation is furthermore supported by studies demonstrating that multisensory integration leads to a more robust percept (Ernst & Bülthoff, 2004), induces cross‐modal plasticity in multisensory conversion zones (Driver & Noesselt, 2008) and facilitates nociceptive neural and behavioural responses, as indicated by invertebrate models (Hu et al., 2017; Ohyama et al., 2015). Therefore, the such a neurobiological mechanism may plausibly explain the observed increase in stimulus‐selectivity to nociceptive cues in healthy individuals performing repetitive movements, as demonstrated in our musician model (Zamorano et al., 2015).

### 4.3 Limitations

The present study has several limitations. First, the effects shown in the current study might likely differ depending on the kind of instrument (Bangert & Schlaug, 2006; Gebel et al., 2013). Future studies should therefore replicate these results in homogeneous samples of participants playing the same instrument and with practice times as a variable of interest. Conversely, however, the fact that musicians in this study belonged to several different instrumental groups (string, keyboard, brass and woodwind) might reflect common consequences of extensive sensorimotor training rather than the specialized instrument‐specific effects. Second, this cross‐sectional study cannot exclude that other confounding factors, such as cognitive processes (e.g. attentional allocation, cognitive appraisals) or inherent pre‐existing factors, which might predispose musicians to enhanced neural responses, may have modulated the pain responses. Therefore, longitudinal studies should be carried out to track the temporal dynamics of pain processing in people performing repetitive sensorimotor training.

## 5 CONCLUSION

The current study described the cortical mechanisms that link extensive sensorimotor training and corresponding multisensory integration to neural adaptations in nociceptive pathways using experienced musicians as an ecological model. Enhanced neural responses to electrical nociceptive and non‐nociceptive somatosensory stimulation in musicians relative to non‐musicians provide the first direct evidence for a link between altered processing of nociceptive inputs and repetitive sensorimotor training in healthy humans. These novel findings may contribute to the understanding of the high variability in neural responses to nociceptive stimulation in the general population and extend current putative models that explain the increased vulnerability for altered pain processing prevalently found in individuals performing repetitive movements. Further neurophysiological research using experimental models of persistent pain is warranted to investigate if these neural adaptations may be considered a risk factor for developing chronic pain.

## AUTHOR CONTRIBUTIONS

All authors provided significant contributions to this manuscript and approved the final version to be published. Anna M. Zamorano contributed to conception and design of the experiment, data acquisition and analysis, interpretation of data, and elaboration of the manuscript; Boris Kleber and Federico Arguissain contributed to conception and design of the experiment, analysis, interpretation of data and elaboration of the manuscript; Peter Vuust and Herta Flor contributed to conception and design of the experiment and elaboration of the manuscript; Thomas Graven‐Nielsen contributed to conception and design of the experiment, interpretation of data and elaboration of the manuscript.

## FUNDING INFORMATION

This work was supported by the Center for Neuroplasticity and Pain (CNAP), which is supported by the Danish National Research Foundation (DNRF121), and by a grant from the Lundbeck Foundation to AZ (R303‐2018‐3356). PV and BK are affiliated with the Center for Music in the Brain that is supported by the Danish National Research Foundation (DNRF117).

## CONFLICT OF INTEREST

None.

## Supporting information


Table S1

Table S2
