# Supplementary material for: Extensive sensorimotor training enhances nociceptive cortical responses in healthy individuals
Source: Eur J Pain. 2022 Dec 1;27(2):257–77. doi: 10.1002/ejp.2057 (PMC10107321; doi:10.1002/ejp.2057)
Supplement: Supplementary file 1 — Table S1 [file EJP-27-257-s001.docx]

| **N200** | **Left FC** | **FCz** | **Right FC** | **Left C** | **Cz** | **Right C** | **Left CP** | **CPz** | **Right CP** |
| --- | --- | --- | --- | --- | --- | --- | --- | --- | --- |
|  |  |  |  |  |  |  |  |  |  |
| **Musicians**  (177± 38ms) | -8.0 ± 6.1 | -9.2 ± 6.1 | -6.1 ± 5.3 | -8.7 ± 6.3 | -9.3 ± 5.9 | -7.1 ± 5.5 | -6.8 ± 4.7 | -6.0 ± 4.9 | -5.8 ± 5.8 |
| **Non-musicians**  (169±30ms) | -4.9 ± 4.1 | -5.2 ± 5.1 | -4.0 ± 3.5 | -5.6 ± 4.1 | -5.3 ± 4.9 | -3.9 ± 4.6 | -4.8 ± 3.1 | -3.4 ± 3.8 | -4.1 ± 3.3 |
| **P200** |  |  |  |  |  |  |  |  |  |
|  |  |  |  |  |  |  |  |  |  |
| **Musicians**  (228±31ms) | 0.8 ± 4.3 | -0.5 ± 5.1 | -0.9± 4.6 | -0.6 ± 4.5 | 0.5 ± 4.5 | -1.5 ± 3.9 | 0.3± 3.6 | 2.4 ± 4.5 | -0.5 ± 3.7 |
| **Non-musicians**  (210±22ms) | 0.9 ± 3.0 | 2.3 ± 3.7 | 1.2 ± 2.7 | 1.3 ± 2.8 | 3.1 ± 3.5 | 0.6 ± 2.6 | 1.6 ± 2.9 | 3.7 ± 3.5 | 1.1 ± 2.7 |
| **P300** |  |  |  |  |  |  |  |  |  |
|  |  |  |  |  |  |  |  |  |  |
| **Musicians**  (369±44ms) | 10.0 ± 5.2 | 13.7 ± 6.2 | 11.4 ± 5.8 | 11.4 ± 5.8 | 17.0 ± 7.2 | 11.4 ± 5.3 | 13.2 ± 5.5 | 20.1 ± 6.4 | 13.8 ± 4.5 |
| **Non-musicians**  (361±60ms) | 9.4 ± 3.5 | 13.0 ± 5.7 | 8.8 ± 3.9 | 11.9 ± 3.8 | 16.3 ± 6.4 | 10.4 ± 4.0 | 13.1 ± 3.9 | 18.2 ± 6.0 | 12.3 ± 4.6 |
|  |  |  |  |  |  |  |  |  |  |
