# Supplementary material for: Extensive sensorimotor training enhances nociceptive cortical responses in healthy individuals
Source: Eur J Pain. 2022 Dec 1;27(2):257–77. doi: 10.1002/ejp.2057 (PMC10107321; doi:10.1002/ejp.2057)
Supplement: Supplementary file 2 — Table S2 [file EJP-27-257-s002.docx]

| **N140** | **Left FC** | **FCz** | **Right FC** | **Left C** | **Cz** | **Right C** | **Left CP** | **CPz** | **Right CP** |
| --- | --- | --- | --- | --- | --- | --- | --- | --- | --- |
|  |  |  |  |  |  |  |  |  |  |
| **Musicians**  (141±10ms) | -9.8 ± 6.4 | -13.0 ± 10.0 | -8.9 ± 6.2 | -10.6 ± 6.8 | -12.7 ± 10.5 | -10.7 ± 7.4 | -9.2 ± 6.0 | -8.8 ± 8.8 | -8.9 ± 7.4 |
| **Non-musicians**  (141±13ms) | -5.9 ± 5.3 | -7.6 ± 7.4 | -5.6 ± 5.6 | -6.5 ± 5.4 | -7.2 ± 7.1 | -6.7 ± 5.3 | -5.6 ± 4.5 | -4.6 ± 5.6 | -5.4 ± 4.1 |
| **P200** |  |  |  |  |  |  |  |  |  |
|  |  |  |  |  |  |  |  |  |  |
| **Musicians**  (197 ± 11ms) | 2.6 ± 4.6 | 4.1 ± 6.1 | 2.8 ± 4.4 | 2.4 ± 5.3 | 6.2 ± 7.2 | 2.4 ± 4.8 | 2.4 ± 5.0 | 7.1 ± 6.1 | 3.0 ± 5.1 |
| **Non-musicians**  (192±11ms) | 5.0 ± 4.8 | 7.1 ± 7.6 | 3.6± 4.8 | 5.5 ± 4.3 | 9.7 ± 7.5 | 3.4 ± 4.2 | 5.9± 3.9 | 9.6 ± 6.2 | 3.8 ± 3.7 |
| **P300** |  |  |  |  |  |  |  |  |  |
|  |  |  |  |  |  |  |  |  |  |
| **Musicians**  (307±26ms) | 13.0 ± 5.7 | 18.0 ± 6.4 | 11.5 ± 4.7 | 16.5 ± 6.8 | 23.0 ± 6.3 | 14.4 ± 4.8 | 17.9 ± 5.7 | 26.2 ± 6.9 | 17.4 ± 4.8 |
| **Non-musicians**  (312±33ms) | 11.7 ± 3.1 | 16.5 ± 6.9 | 10.6 ± 4.6 | 13.9 ± 3.3 | 20.4 ± 7.6 | 12.2 ± 4.6 | 15.8 ± 3.9 | 20.9 ± 5.2 | 14.1 ± 4.3 |
|  |  |  |  |  |  |  |  |  |  |
